# Root Canals and Conspiracies: A Social Semiotic Analysis of Digital Narratives on Social Media and the Promotion of Misinformation

**DOI:** 10.3390/dj13100453

**Published:** 2025-10-02

**Authors:** Alexander C. L. Holden

**Affiliations:** 1 Sydney Dental Hospital and Oral Health Services, Sydney Local Health District, Sydney, NSW 2010, Australia; alexander.holden@sydney.edu.au; 2 Sydney Dental School, Faculty of Medicine and Health, The University of Sydney, Sydney, NSW 2010, Australia

**Keywords:** root canal treatment, misinformation, endodontics, semiotic analysis, conspiracy, qualitative research

## Abstract

**Objectives:** Narratives related to root canal treatment on social media can be problematic. This research examines discourses relating to root canal treatment on the popular platform Instagram. Through this analysis, the way different health discourses relating to endodontics are being communicated to the public and consumers will be explored. **Methods:** A search was conducted on Instagram to access consumer-focused information related to root canal treatment. Posts were identified, transcribed to capture the audio component in text form, and watched cyclically, with analytical memos being kept on video content. A social semiotic analysis was conducted on posts to capture the multimodal nature of discourse on endodontics. **Results:** A total of 100 Instagram posts were included in this study. Two overarching themes were used to structure the analysis of posts: (1) presentation of root canal treatment and (2) presentation of expertise. The majority of posts were negative in tone towards root canal treatment. **Conclusions:** The variety and nature of the misinformation featured in this research present a complex professional challenge for dentistry. The majority of the posts identified and analysed featured sensationalised explanations of dental disease and treatment processes, blended with inaccuracies, many of which were produced by dentists. Conspiracy discourses relating to root canal treatment were also encountered, which questioned the integrity and intentions of the dental profession.

## 1. Background

Discourses relating to root canal treatment are varied. Some narratives espouse the benefits of endodontic treatment and its long-standing history of effectively maintaining teeth in the mouth that would otherwise need to be extracted [1]. Other discourses promote a narrative that root canal treatment is harmful, asserting that this therapy leads to an accumulation of bacteria and their byproducts within the body.

The dental profession has strongly advocated that root canal treatment is safe. As one example, the American Association of Endodontists has issued both patient [2] and profession-focused [3] resources that outline the safety and efficacy of the procedure. This discourse, widely accepted as being supported by the established evidence base, meets opposing narratives from minority professional groups in dentistry and wider society. As one example of this, a society of biological dentists, the International Association of Oral Medicine and Toxicology, states on its website: “There is controversy once again in the public’s consciousness over root canal treatment. The origin lies in the question of remnant populations of microbes in the dentinal tubules and whether or not endodontic techniques adequately disinfect them or keep them disinfected” [4]. This narrative speaks to the theory of focal infection, which posits that bacteria from a tooth will spread to distant locations in the body. This was a popular professional explanation in the early 20th century that fell from mainstream evidence-based dental practise due to the lack of scientific basis [5].

The perception that the dominant evidence-based narrative is unquestionable in its legitimacy is excusable, especially when supported by the majority of the profession. However, whilst acknowledging the original focal infection theory’s over-simplicity, the linkage between oral infection and systemic health has revived to some extent of professional acceptance [6]. Similarly, academic commentary on endodontic medicine is modest when reviewing the evidence base on the potential for root canal infection and periapical infection to have an effect on systemic health [7], giving partial legitimacy to those concerned about the risk of persistent infection in root canal treatments. Recent advances in understanding the impact of anti-resorptive medications on the body’s response to infection show that recurrent infections from failed root canal treatments can indeed lead to occult necrosis and infections within the jaw [8]. Albeit different in context from other claims, this shift in understanding—based on changing healthcare treatments and trends—demonstrates how the nature of truth in healthcare evidence-based practise shifts over time. It is therefore important not to dismiss any discourse out of hand, merely because it falls outside of the accepted and dominant view of current professional practise. Contemporary research considering alternate truth discourses advocates for an inclusive approach to those promoting contrary discourses that reduce the risk of further isolation and stigmatisation [9].

The potential for health narratives that are unsupported by evidence to impact healthcare decision making and, therefore, health outcomes is the driving concern in addressing health misinformation. Social media has an acknowledged role in the spread of health misinformation [10], with the nature of platforms allowing fast diffusion and spread of discourses: “It is the capacity for the viral spread of disinformation and doubt that is a pervasive feature of the current media ecology” [11]. Instagram is a widely used platform for user-generated content, with posts of images and short video stories giving a rich source of data for analysis. Instagram is also widely used by influencers to share content on health and well-being, making it an ideal site to examine the variety and nature of extant discourses that relate to root canal treatment.

Content creators on social media can use platforms, such as Instagram, to develop their identities as microcelebrities. Senft defines microcelebrity as “A new style of online performance that involves people ‘amping up’ their popularity over the Web using technologies like video, blogs and social networking sites” [12]. This research will examine how influencers, especially those who may have professional obligations as dentists, utilise social media to promote or disparage root canal treatment as a modality of care. Instagram has been used as a site for health information research in dentistry before [13,14,15,16,17]. Previous research has typically utilised content analysis and has used structured measures of information quality, such as DISCERN, to evaluate the potential for social media information to mislead patients and the public. This research will take a different approach, with this being the first instance of a multimodal analysis of social media information in dentistry. This in-depth analysis, carried out on root canal treatment information, will add value to the academic literature by providing insights into the engagement strategies employed by influencers, as well as the nature of the discourses being promoted.

This research will establish how discourses on root canal treatment are presented and situated on Instagram. Through demonstrating the way that these different narratives are positioned to the public, this research will investigate how influencers (as microcelebrities) are able to frame their health and dentistry-related content to enhance engagement. Finally, the potential implications of discourses on root canal treatment on trust in dentistry will be considered and explored.

## 2. Methods

The reporting of this study follows the SRQR reporting guidelines [18]. The use of publicly available social media content is not without ethical consideration. The public nature of some social media content would not justify the use of the data in research without consent, as to do so would be a breach of the confidentiality and privacy of the original poster. An example where this might be the case would be an instance of a researcher gaining access to a private social media group, then using data encountered within the group for research purposes. It would be reasonable to argue in such an instance that the original poster would not have anticipated, or wished for their posts to travel outside the original venue of posting or be used for a different purpose. Other social media is more public and intended by the poster to be spread far and wide to disseminate a particular message. In the case of this research, the case-by-case approach of assessing the ethics of using social media content was followed, advocated by the Association of Internet Researchers [19] and further developed by Harrington [20] in the context of social media users posting on sensitive topics. Using these principles of assessment, institutional ethics approval was not applied for in relation to this work. This is justified due to the analysis involving publicly available social media content, posted by individuals or groups who were actively promoting professional and lay messages designed to be consumed by a wide audience.

Instagram was searched in June 2025, using the hashtags #rootcanaltreatment and #rootcanaltherapy, as well as the search posing the question “should I have root canal treatment?”. The first 300 results from each search were reviewed to ascertain alignment with inclusion/exclusion criteria. The objective of the search was to identify up to 100 individual posts that met the inclusion criteria to form a corpus of visual and textual resources for analysis. The results from each search were screened for alignment to the inclusion/exclusion criteria in the order in which they appeared. The inclusion of this number of posts was sufficient to achieve saturation for the analysis, ensuring that the analysis captured the most important semiotic aspects of posts. This also provided insights into how both the detractors and supporters of root canal treatment present information, enabling insights to be drawn into the commonalities and differences in the nature of the information provided.

Different formats of posts were included, with video, informatic, and text-based posts being accepted within the corpus. Posts were included if they referenced root canal treatment either as the whole or part of their content and needed to be directed at providing information to a public audience about root canal treatment. Posts that were exclusively directed at dental professionals (i.e., those discussing professional surgical or non-surgical techniques or equipment reviews) were excluded from the corpus. Only posts in English (text or audio) were included. After posts that met the inclusion criteria were identified, textual components were documented, and the audio component of the included videos was transcribed. This created a text-based corpus for analysis, accompanying the visual and semiotic resources provided by the posts. Other information that was captured included the number of followers of the post authors, as well as the number of likes, comments, and shares each post had attracted.

The analysis took a multimodal approach in order to capture the full semiotic nature of the included texts. As the posts included within the analysis represented a complex mixture of visual and text-based media, a methodological approach was needed that would allow the rich interweaving elements of the posts to be included within the analysis in a way that fully recognised the messaging of each post [21]. Employing the social semiotic framework of visual analysis developed by Kress and Van Leeuwen for this purpose [22] allowed all aspects of the posts to be reviewed and analysed. This methodology has been shown to be an effective approach to examining professional representations in oral health, with the gathering of insights into professional identity and relationship to the public being possible through its employment [23]. Multimodal discourse analysis, such as Kress’ and Van Leeuwen’s social semiotic approach [22], is particularly well aligned to research analysing content from social media, which includes multiple types of content (i.e., video, audio, and text). The intent of this largely inductive methodological approach is to draw out rich insights from the data, allowing for detailed analysis of the social and cultural phenomena encountered.

The posts were viewed multiple times, with video content being watched cyclically, with analytical notes being taken on the visual aspects of the content to allow for aspects of the semiotic resources within the texts to be captured. In examining the multitude of ways in which root canal treatment is presented and discussed on Instagram, attention to discourse becomes of key importance. Discourse has been defined as follows: “Socially situated forms of knowledge about (aspects of reality): This includes knowledge of events constituting that reality (who is involved, what takes place, where and when it takes place, and so on) as well as a set of related evaluations, purposes, interpretations, and legitimations” [24].

Through discourse analysis, the interactions between the actors within the visual texts analysed can be examined and explored, with insights into their intended relationship and impact on the consumer being discoverable. As a multimodal methodology, social semiotic analysis incorporates discourse analysis into the overall examination of visual and text components of the data. Social semiotic analysis categorises three different types of semiotic work that occur together and simultaneously within visual media. Each category can be described as a particular metafunction: (1) the representational metafunction; (2) the interactive metafunction; and (3) the compositional metafunction. These metafunctions and their relevance to the corpus are described in more detail in Appendix A.1. The metafunctions give structure to the consideration and discussion of the visual components within texts and, within this analysis, allow for a systematic examination of their contributions to the discourse within the corpus. This research was conducted by ACLH, an academically trained dentist and specialist in public health dentistry.

## 3. Findings

### 3.1. Description of the Corpus

The corpus was comprised of 100 individual posts from Instagram identified by the search strategy. These posts were created by 72 different Instagram accounts, with almost a quarter (23 posts) being from just three creators who are dentists. Within the corpus, 4 posts were infographic in nature, 3 were multi-slide carousel images, 5 were photographs with captions, a single post was purely text-based, and 87 were videos.

### 3.2. Sentiment Towards Root Canal Treatment

75 posts had a negative tone towards root canal treatment, with 21 being positive in tone and 4 being assessed as neutral in attitude towards root canal treatment. Posts were deemed to have a negative tone if their content actively dissuaded having root canal treatment, due to claims of the treatment being harmful. Those posts determined to have a neutral tone presented objective statements in relation to root canal treatment, with those deemed to be positive actively encouraging those who have identified a need for treatment to have this provided.

### 3.3. Nature of Content Creators

62 of the posts were made by dentists, with 20 being from alternative health promoters (other health professionals) and 18 being from non-health professional health influencers. 38 posts made by dentists were negative towards root canal treatment.

### 3.4. Nature of Negative Posts

Within the 75 posts that were negative towards root canal treatment, all were classified as misinformation, as all claimed root canal treatment was harmful and should be avoided. These posts were coded in the following ways: (1) alternative treatments promoted (10 posts); (2) conspiracy theories (10 posts); (3) health risks exaggerated (10 posts); (4) misuse of outdated research (1 post); and (5) scientific misunderstanding (44 posts). Some posts met more than one codifying theme but were categorised in alignment with the dominant theme of the post.

### 3.5. Engagement with Posts

Posts that had negative sentiment had, on average, 13,987 likes and 399 comments, compared to an average of 2295 likes and 307 comments for posts with positive sentiment.

### 3.6. Social Semiotic Analysis

Through watching and rewatching the post video content and reviewing the post transcripts, notable themes emerged from the corpus. These themes were developed and refined over iterative rounds of coding. The themes and sub-themes are displayed in Table 1. The findings are presented by theme, accompanied by an examination of how semiotic resources have been applied to the content. Examples from the corpus are used to illustrate how the themes have developed and are presented within the research. Examples are identified as individual posts, so the spread across the corpus is transparent. The number of likes (👍) and the number of comments (💬) for each post are provided to indicate the level of engagement each post has attracted.

### 3.7. Presentation of Root Canal Treatment

#### 3.7.1. A Flawed Treatment

Posts in the corpus provided a high level of detail on how root canal treatment represented a threat to health and why this was a fundamentally flawed clinical methodology. The posts that negatively portray root canal treatment employed emotive language, such as teeth having had the treatment being ‘dead’, and that this was an inherently bad idea:

*Here’s the truth, a root canal leaves behind a dead tooth*—Post 2 (👍 1529 💬 33)

*(I)t has never been a good idea to leave a dead organ in your body*—Post 4 (👍 449 💬 84)

Posts were also clear in articulating why having a dead tooth retained in the body was a problem:

*The most dangerous thing you can have in your mouth is root canals, and the reason for this is a root canal filling is a dead tooth. The root has been taken out so there is now no blood, no lymph going through that tooth to clean it. Microbes can live in there*—Post 21 (👍 27.1 K 💬 584)

*(T)he dead tooth becomes the perfect cave for anaerobic bacteria*—Post 23 (👍 316 💬 24)

A common thematic presentation of the root canal treatment process by those averse to the treatment is that the therapy is ineffective due to resulting in chronic infection that will spread to the jaws:

*I call the root canal procedure a fatally flawed procedure. It’s not flawed in the sense that it won’t relieve pain. As I said, you take out the nerve and blood supply, you’ll often eliminate the pain. But you absolutely assure, even if it wasn’t present before, that you’ll always end up with a chronic infected tooth. And when it’s in the molar area and you chew on it with the enormous pressures that the jawbone can generate you push those pathogens and toxins into the bloodstream*—Post 25 (👍 546 💬 99)

The importance of discourses on the nature of root canal treatment is emphasised by how the nature of causation and correlation between root canal treatment and other aspects of health is explored within the corpus, and this is developed further in the next theme.

#### 3.7.2. Causation vs. Correlation

Chronic infection in root canal-treated teeth was presented as an inevitability, asserting that this would precipitate other health issues, stating that there is evidence for this claim within peer-reviewed literature:

*Believe it or not, in the dental literature, a survey that shows patients that have one or more root canals in their mouth, not failed root canals, not poorly done procedures; they just have a root canal in their mouth. They have a greater chance of heart disease and heart attack. People need to be told this before they get the procedure*—Post 25 (👍 546 💬 99)

The corpus included discourses of a causative relationship between root canal treatment and a number of significant systemic health conditions, including cancer:

*What if I could tell you that 98% of women that have breast cancer have a root canal tube on the same side as their offending breast cancer? Of the people that come to see with chronic illness, the question is how many of ‘em have a dental aetiology to the cause of their illness? I would say almost all of them*—Post 36 (👍 1055 💬 40)

*There’s a Switzerland clinic…went back 20 years, 98% of their breast cancer patients had a root canal on the same tooth as the initial tumour. I’ve taken hundreds of these that we extract the tooth we send it to the laboratory, find out what’s in it, and we get back sometimes with 30 different strains of pathogenic bacteria. Parasites. We find some crazy stuff in these root canals that are just slowly leaching out into your system*—Post 8 (👍 11.4 K 💬 453)

One post did acknowledge that causation and correlation were separate matters; however, the post demonstrated a strong belief that correlation between having both a root canal treatment and a systemic illness was evidence of root canal treatment’s role in causing ill-health:

*I’m not going to say and I wouldn’t begin to say that there aren’t a lot of people out there with root canals that are doing fine, but I can tell you flipping around almost all of the ones that aren’t doing well have the root canals too*—Post 25 (👍 546 💬 99)

#### 3.7.3. Sensational Information and Language

Alongside the use of emotive words such as ‘dead’ and ‘toxin’, content creators frequently referred to the presence of parasites within teeth that had been root canal-treated. While the term parasite could legitimately be used to describe any exploitative relationship between a pathogen and a host (covering any number of bacterial, fungal, or viral infections), in some of the videos, this narrative was accompanied by AI-generated visual cues of larger complex parasites, such as insects and worms, writhing inside a tooth. This serves as an example of how some posts within the corpus would take evidence-based information and sensationalize through the use of emotive language and imagery, and then blend with misinformation through spurious or misleading inferences. As a semiotic tool, the representational metafunction is used to highlight the intended meaning that the video’s narrative gives about the nature of root canal-treated teeth being purportedly infested.

When this is then coupled with mistaking correlation for causation (i.e., 98% of women having cancer treatment also had a root canal treatment), the mixture of science, pseudo-science, and misinformation becomes harder to evaluate.

One dentist content creator drew alignment between the outdated and antiquated nature of root canal treatment in dentistry and its safety:

*Are root canals even safe anymore? We really have to rethink conventional root canal therapy. What’s promising on the horizon is stem cells, the regrowth of teeth regenerating pulps with stem cells. The pulp of the tooth, the center of the tooth, regenerating that, something called pulpal regenesis, and that is the future. Not doing embalming or taxidermy on a tooth the way old root canals used to be done*—Post 78 (👍 717 💬 92)

The way that supporters or proponents of root canal treatment present the procedure is less extreme and uses softer language than those who claim that root canal treatment is unsafe or ineffective:

*So what we do is we dress the tooth with a nice little plastic sheet. And we open the tooth up, remove all the bugs and decay clean down into the roots of the teeth…And then we put something else in there, it fills the gap and stops new bugs getting back in. Most of the time with these teeth, if it’s a back tooth, we put a little crown on it or an onlay, or if it’s a front tooth, we put a little filling on it…Afterwards, it can be a bit sore, but otherwise, generally fine. It settles down*—Post 66 (👍 36 💬 1)

The use of language such as “nice” and “little” presents root canal treatment in a less confronting way, as well as acknowledging the poor reputation that the therapy has as being painful:

*The biggest thing I sometimes get asked is, is this painful? And I can guarantee that most of the time is absolutely not. You won’t even know that it’s happening other than you’re a little bit bored and you sat in the dental chair for a very long time*—Post 66 (👍 36 💬 1)

Other posts also focused on concerns over the representation of root canal treatments as painful treatments:

*This is the truth about root canals that no one talks about. Send this to someone who’s been putting off a root canal, out of fear. They’re not the nightmare you’ve heard about. Thanks to modern tools, they’re faster, cleaner, and way more comfortable than before. Most patients say it feels like getting a regular filling, usually done in one visit*—Post 72 (👍 338 💬 29)

Some of the posts supporting root canal treatments were combative towards different perspectives relating to the treatment, demonstrating frustration at the suggestion that root canal treatment is unsafe:

*And here I am looking at Instagram and I’m just a little tired listening to some of the myths that are getting propelled out there for people to listen to. So let’s unravel some myths right now…when root canals are done properly, when the canals are clean, shaped and filled… when they’re filled properly, those areas that apex the end of the root does not leak. So you don’t have what’s called an inflammatory response. They do extremely well*—Post 48 (👍 165 💬 11)

*No, that’s bullshit. Root canals don’t cause cancer. There’s no scientific evidence to back up this claim, and if anything, if needed, it’s a great treatment option to prevent you from taking the tooth out and replacing it with an implant*—Post 64 (👍 60 💬 8)

One creator, an endodontist in support of root canal treatment as a therapy, made disparaging comments about colleagues’ ability to perform the treatment effectively:

*When root canals are done without CBCT and microscope, THEY FAIL. Say NO if your dentist doesn’t have enough experience to do your root canal*—Post 53 (👍 399 💬 20)

Some proponents of care, as well as those who had hesitance over the treatment, tried to take an approach that, whilst robust, also acknowledged different perspectives in answering whether root canal treatment is safe, even though this discourse results in suggesting that there is a possibility of death for those avoiding endodontic treatment:

*Are root canals bad for you? I know there’s a lot of controversy about it. I will tell you this. If you need a root canal and you don’t do it, that’s really bad for you. In fact, the only part of the tooth that can die is a nerve…when that nerve dies, it becomes necrotic and it causes an infection in the bone. So if you don’t treat that, you can have a massive infection. In fact, you can actually end up in the hospital and in the worst, worst, worst case scenario, you actually can die from an infected tooth. So if you have a dead tooth, you have two choices. Either you extract the tooth into an implant. Or you go ahead and do a root canal, and I would highly recommend you do a root canal…you will not die from a root canal. You might die if you don’t do a root canal*—Post 34 (👍 18 K 💬 57)

While the advice from the following post gives hallmarks of being derived from a supporter of biological dentistry, it stops short of the advice that many from this field give of extraction and replacement of teeth that have received root canal treatment:

*If you’ve had a root canal, here’s what I need you to know, as a dentist of 40+ years: (1) schedule a CBCT scan every 3–5 years, (2) track your CRP, (3) root canal treatments can last decades or longer, (4) you should monitor it just like any implant in your body, (5) bookmark this and ready the caption for detailed advice*—Post 28 (👍 3553 💬 1384)

The presentation of root canal treatment in the corpus is inconsistent, ranging from the treatment being life-saving and part of routine dental care to being an unsound, outdated, and dangerous treatment modality.

### 3.8. Presentation of Expertise

#### 3.8.1. Evidence-Based Arguments

Dentists promoting endodontics as a viable, effective, and safe treatment option quoted the existence of scientific literature to support the treatment and also offered those engaging with the possibility of discussing with members of the profession:

*So there is no evidence-based research proving any association between root canal treated teeth and cancers and tumors. For more information, you should talk to your endodontist. Your endodontist would be more than happy to share evidence-based research and studies to debunk the myth*—Post 70 (👍 36 💬 1)

Some of the creators within the corpus would actively refer to the existence of published peer-reviewed literature in their commentary against root canal treatment (an example of this has also already appeared in the previous theme):

*We know there’s studies that show on the side of a root canal is an increase in breast cancer on the side of a root canal*—Post 76 (👍 48.8 K 💬 889)

*Well, it sounds like there’s a lot of things going on that we’re not seeing. Yes. To your point, there is a lot of pushback of dentists who say, oh, I don’t believe that. I don’t believe that. No, they’ve done studies. There’s PubMed studies. You can go online, 100% of endodontically treated teeth, root canal teeth produce endotoxins, which are bacteria and bacterial byproducts*—Post 55 (👍 4333 💬 443)

This post also suggests a hidden nature to some knowledge that the majority of dentists are not prepared to accept. Another post went further and directly quoted from published literature from the *Journal of Endodontics*:

*“DNA analysis of extracted root canal samples from treatment-resistant cases found microbes known to trigger systematic inflammation in 100% of patients”—Journal of Endodontics, 2016. PMID: 27377440*—Post 73 (👍 695 💬 150)

The reference to hidden knowledge links this theme to the next theme, which discusses the nature of the relationship between powerful stakeholders within the corpus. While all misinformation involves the promotion of false and misleading information, conspiracy theories emerged within the corpus as a particularly visible category of misinformation. There is a suggestion of conspiracy by the dental profession and commercial interests, either overtly or covertly, throughout the corpus.

#### 3.8.2. Commercial Conspiracy

Misinformation is presented as established knowledge within the corpus. Theories of conspiracy, between the dental profession and commercial interests, are also frequently encountered, especially in reference to a film that was withdrawn by Netflix about root canal treatments, Root Cause, which is directly mentioned in 11 posts. The profession’s lack of action on root canal treatments is linked to commercial conflict of interest by one dentist influencer:

*You are telling me that this is like a billion dollar business? No, no, no. Trillions. Okay. Trillion dollar business. It’s about $3000 for, you know, two to 3000 for one root canal. So why now multiply that by 25 million…so why would they want this to go away if it’s a trillion dollar business?*—Post 55 (👍 4333 💬 443)

Later, in the same post, the dental profession’s altruism is questioned:

*Sounds like they would rather make the money than care about the inflammation in your mouth, which is linked to multiple diseases*—Post 55 (👍 4333 💬 443)

In multiple posts from different creators, the same video clip of influencer Luke Belmar is replayed and referenced. Belmar is an entrepreneur who is well known for supporting anti-establishment ideologies and promoting topics such as cryptocurrency and online enterprise. In the referenced clip, Belmar is being interviewed and is asked to talk about a conspiracy theory that he 100% believes is true. His answer:

*Root canals—There’s this YouTube video which before was a documentary on Netflix before it got pulled after 2 weeks known as the Root Cause. The Root Cause discusses the big psyop by dental industry and big pharma to fuck people’s health up through root canals. You can’t even watch it in the United States, you have to turn on a VPN because it’s banned by the American Dental Association which is in bed with big pharma. I had a root canal recently removed and once they did the DNA biopsy on the tooth, they found 20 different types of parasites growing and living inside of my jaw, which is absolutely horrendous, and obviously the dentist isn’t going to tell you that, stay away from fluoride and stay away from root canals*—Post 1 (👍 189 K 💬 2722)

Other narratives that relate to Root Cause and conspiracy are also present in the corpus:

*I know that there was a documentary on Netflix. It was called The Root Cause…and it exposed a lot of things that you’re talking about, and it educated the population on a lot of things that you’re talking about. And then it mysteriously disappeared. It’s gone. It was a class action lawsuit by the American Association of Endodontists, and they said it was scaring the public or something ridiculous…they’re protecting the interests of their members and their members…they make their living doing this stuff*—Post 86 (👍 369 💬 18)

*Have you guys seen the movie root cause? So 100% of root canal teeth are infected out of thousands and thousands and thousands of teeth. They tested 100% of the teeth are infected*—Post 6 (👍 4913 💬 211)

The posts speak of hidden knowledge that has been kept that way due to the interference and curation of powerful professional interests that are numb to the needs of consumers.

While the dentists who oppose root canal treatment are quick to suggest commercial self-interest from dentists promoting root canal treatment, there is no recognition that the same allegation could be made in relation to their own promotion of ceramic implants as an alternative treatment option and the optimal solution for addressing tooth replacement:

*Get it out. The tooth is dead. Don’t leave it in there and get a zircon Implant. Don’t let them put a titanium in there. If they put a titanium implant in there, which they say is stronger and better is not, it’s a metal that’s in your body now. You have a chunk of metal that is radiating metal energy of whatever sort on that meridian. That’s why zircon is so important ‘cause that’s ceramic and they’re strong. They’re a little bit more money, but they’re worth it*—Post 85 (👍 14 K 💬 469)

*A potential solution for a chronic infected root canal is an immediate ceramic implant which all the other reels about it in my feed*—Post 6 (👍 4913 💬 211)

#### 3.8.3. Personal Risk

Linking with the idea of elite professional and commercial interests controlling practise in dentistry, posts in the corpus highlighted the personal risk that they and other dentists took in having a stance that was contrary to the rest of the profession:

*Dentists can’t say it because they’ll lose their license*—Post 43 (👍 416 K 💬 438)

One post, featuring an interview with popular influencer Luke Belmar, highlighted the advertised risk of speaking out on any aspect of dentistry that did not agree with mainstream practise.

*I’ve talked to dentists that are bio dentists that have had mafia level pharma come to them saying, Hey, we’re gonna send patients in. And if you tell ‘em that Mercury poisons your body, we’re taking your license away. We’re taking you to jail*—Post 31 (👍 553 💬 205)

One of the dentist content creators, whilst they did not speak of any concerns related to persecution for their professional beliefs or practise, did speak to the fact that they no longer supported or provided root canal treatment now:

*I was a big fan at one time*—Post 10 (👍 27.2 K 💬 1454)

This demonstration of having been part of mainstream, accepted dental practise and then having turned away from this helps to support the legitimacy of these content creators’ expertise.

#### 3.8.4. Expertise and Engagement

The representation metafunction within semiotic analysis is concerned with who and what is represented within the visual text. Within the corpus, differences in the presentation of information and expertise are already apparent just from the analysis of texts; however, social semiotic analysis also considers the other, visual aspects of the posts that make up the different components of the corpus.

All health providers who appear in the corpus (i.e., dentists, naturopaths, chiropractors, and others) leverage their professional status to amplify their expertise. Aside from their clinical messaging, clinical expertise was signified through professional dress (many wearing scrubs) and often filming within their clinical offices/surgeries. Notably, within the corpus of the professional content creators who identified as dentists, there was no discernible difference in this strategy of dress and filming location between those who supported and those who opposed root canal treatment.

The main difference between the two professional dental groups is the standard and level of video production within the videos they generate. The content where dentists support root canal treatment is often produced by practitioners posting as part of an advertising and patient engagement strategy to support their clinical dental practice, which is their primary purpose. The videos in themselves are not a product; instead, they are an engagement tool to help build a community around their patient base. In contrast, influencers are often posting content as a primary objective in promoting themselves and monetizing their social media content. The nature of the content is therefore designed differently from those promoting a practice or seeking to engage with a far smaller local community, where engagement beyond that immediate social group has little benefit or value. Videos posted by those promoting an antagonistic stance towards root canal treatment typically had far higher production values, with engaging background music and integrated graphics and breakout videos within the video post. This contrast is evident in the levels of engagement seen within the videos.

The interactive metafunction examines how the viewer is positioned to interact with the media content. This focuses on the distance from the subject of the video and the camera, the angle of the camera that the video is taken with, along with other mechanisms of the way that the video is produced, in order to structure how the viewer engages with the content. Influencers within the corpus had a far better understanding of how intimacy and a relationship with the viewer might be developed. Dentists promoting root canal treatment typically did not demonstrate awareness of semiotic tools and mechanisms, with videos being shot with the dentist presenting being further away from the camera than influencers might be, encouraging feelings of distance, emphasizing the traditional professional position of being detached from consumers (patients). Influencers, both dentists and others, utilised visual grammar to better foster engagement. Influencers were more likely to utilise close-up camera angles, also engaging strategies to build intimacy, such as shooting candid videos, where influencers appear to be speaking more directly to the viewer, with a more personal feeling of engagement.

The compositional metafunction, which serves an overarching role, brings the representation and interaction metafunctions together to give the overall meaning behind the visual text. The more professionally focused, clinical content of dentists speaking positively about root canal treatment has an overall detached nature, with distance between the viewer (encouraged to take on the role of patient) and the information being transmitted without drama or effect. The presentation of positive discourses on root canal treatment is typically over-reliant on the perceived value and clinical authority of the information to support its delivery. In contrast, influencers have the ability to shift the relationship between themselves and the viewer to being more personal, confiding, and special, as if the viewer is part of the secret that the influencer is sharing. This increases the likelihood of engagement with this media, with viewers being more encouraged to interact with the content of the texts. Examples of how the metafunctions apply practically to two of the posts can be found in Appendix A.2 and Appendix A.3.

## 4. Discussion

### 4.1. Dentist vs. Dentist: Dualling Expertise Within the Corpus

Within the corpus, many of the analysed texts that warn against root canal treatment have been produced by dentists. It is of significance to have members of the dental profession actively promoting against an established treatment modality that is supported by a broad base of supporting evidence and a history of clinical success. Exploring this phenomenon is important in the context of better understanding how those who might promote and spread misinformation in dentistry view themselves and their role interacting with the profession, either as professional or non-professional stakeholders. Hofstadter articulates the appeal of spreading misinformation and conspiracy; through embracing conspiracy narratives, individuals become “a member of the avant-garde who is capable of perceiving the conspiracy before it is fully obvious to an as-yet unaroused public” [25].

This research has shown that many narratives that can be considered to be misinformation are being driven and promoted by dentists. Baker explores the powerful effect that espousing conspiracy theories and misinformation that is contrary to accepted belief has upon experts: “a conspiracy theory is an invitation to join an enlightened but embattled minority—an elect few who bravely, selflessly speak truth to power” [26]. Within the corpus, there is evidence of either a reality or a belief of persecution and stigma in relation to the role of being a dentist who stands against the practise of root canal treatment. Without conspiracy theories, with ideas that are non-evidence-based or disproved, these alternative experts become plainly wrong. Through conspiracy theories and misinformation, alternative experts “elevate sensory experience, bodily wisdom and intuitive ways of knowing as superior modes of epistemic reasoning over abstract facts and knowledge” [11].

The negative content that is produced by dentists is produced by a relatively small number of individuals who have posted multiple times. This mirrors observations made during the COVID-19 pandemic that a small group of twelve influencers was estimated to have generated almost two-thirds of anti-vaccine content during the COVID-19 pandemic [27]. The professional dentist influencers who espouse alternative beliefs and misinformation related to root canal treatment provide a blend of professional legitimacy and half-truths that may be hard for consumers to evaluate for the reliability of their content. In some instances within the corpus, alternative experts refer to cherry-picked components of scientific literature to support their own non-evidence-based claims as a way of furthering the legitimacy of their position. A prime example of this is the direct citation of a paper from the Journal of Endodontics that purports to show the presence of harmful bacteria inside root canal systems [28]. The reality is that this cited research considers the microscopic ecosystem of a root canal system in cases where endodontic treatment has failed; in such an instance, it would be unusual not to find bacteria of significance within the root canal space. The corpus included multiple instances where claims were made relating to root canal treatments causing breast cancer. There is currently no accepted high-quality evidence that supports this claim [29].

Those within the corpus who support the accepted evidence-based position related to root canal treatment are hard to differentiate from their alternative colleagues in regard to legitimacy and the semiotics within their content. In almost all cases within the corpus, non-conforming dentists presented content with higher levels of engagement than those dentists promoting the safety and efficacy of root canal treatment. Part of the reason for this higher level of engagement is that those promoting misinformation and conspiracy theories harnessed “awareness, position in conventional and social media, to offer emotional support, an identity matrix and pedagogy for self-discovery and well-being” [30]. The dentists and influencers sharing negative sentiment towards root canal treatment were far better at utilising semiotic tools to leverage their higher engagement. Egalitarianism, authenticity, access, and autonomy are defined by Baker and Rojek [30] as common strategies utilised by influencers to heighten intimacy between themselves and their viewers. Within the corpus, these tools of engagement are used semiotically to boost the reach of messaging, through camera angles, music, and blending professional and personal narratives and contexts to create high impact content.

In comparison, those dentists promoting root canal treatment demonstrate an over-reliance on consumer regard and deference to evidence-based practise and professional position to legitimise their messaging, with the semiotic potential of posts to heighten engagement being largely unrealised. The self-presentation strategies of those promoting root canal misinformation assist in the transfer of trust from traditional sources of medical and health authority to these alternative sources of knowledge and understanding [31]. Further content legitimacy is built by dentists promoting misinformation through their promotion of the perceived professional risk they take in promoting their beliefs. It is common for these content creators to reference that they may face repercussions from the rest of the profession for speaking out, which is presented as commercially conflicted and vindictive.

### 4.2. How Is the Dental Profession Helping to Drive Conspiracy and Misinformation Narratives?

Notorious conspiracy theorist, David Icke, outlines his concerns with traditional institutions within society as, “Everything is backwards; everything is upside down. Doctors destroy health, lawyers destroy justice, universities destroy knowledge, governments destroy freedom, the major media destroy information and religions destroy spirituality” [32]. The belief in a hidden elite within society who are actively damaging the population’s health and well-being is common within conspiracy narratives, Baker states, “At the heart of conspiracy theories is narrative storytelling, in particular plots involving influential elites secretly colluding to control society” [11]. Icke is notable in his extreme theories and views on the order of our society, and yet one of the challenges that conspiracy theories present is the crossover from fiction to reality that the concerns related to professional behaviour and intent represent.

It is apparent in the corpus that those who advocate against root canal treatment do not advocate against dentistry in its entirety. Harambam notes that conspiracy theorists have not lost faith in the totality of traditional institutions, but these entities are believed to no longer fulfill their purpose, echoing Icke, he states, “religion does not inspire spirituality, politics does not create a better world, science fails to establish truthful knowledge, and the media are not a reliable news source” [9]. To those believing conspiracy theories, these institutions have lost their true meaning due to corruption from external factors. When considering this narrative within dentistry, we see, too, that the dental profession struggles with its relationship to commercial factors; distrust in the profession is not without justification. There is a growing body of research that highlights the negative impact of business considerations on dentists’ behaviour, with commercialism being an intrinsic consideration to viable practise, influencing business and treatment decisions [33,34]. While dentists may embrace social media as a tool for oral health promotion, many of the videos produced promoting root canal treatment were consciously and overtly branded to promote a dental practice where that treatment might be accessed.

It is therefore not difficult to empathise with those who might suggest that the dental profession has been unduly influenced by ‘big pharma’ to promote a treatment modality that causes harm, even though we might reject the premise of that particular accusation in the case of root canal treatment being harmful and performed simply for dentists’ financial gain. Within the corpus, mainstream professional knowledge and treatment approaches are presented by those who are critical of root canal treatment as being commercially controlled and therefore compromised, with alternative information being untarnished by commercial interest. It may be that before the dental profession is able to dismiss what may appear at first instance to be outlandish claims of commercial interference with treatment decisions, the professional community needs to address its complicated relationship with commercialism more broadly. The dentists within the corpus who promote conspiracy theories and misinformation related to root canal treatment are not without their own commercial conflicts of interest. Many of the videos analysed, which featured dentists promoting anti-endodontic narratives, promoted alternative, often more expensive dental procedures, such as ceramic implants and bone grafting, as solutions to having endodontically treated teeth removed.

### 4.3. Limitations

This research is bound to the time, space, and venue where it was carried out; it may be that the data, findings, and inferences gathered and produced by other researchers would yield different insights from this research inquiry. Similarly, this research relates to narratives found on one social media platform, and care must be taken in making any generalisations to other social media contexts, or to content produced in languages other than English. Nevertheless, this research is presented in a manner that allows transparency in how conclusions have been arrived at and positioned [35]. This work claims analytical modesty in the insights derived from the data included [36]. These limitations do not detract from the value of engaging in this type of research inquiry and do not discount the conclusions drawn. The value of this research is that both the data and the researcher contribute to the synthesis of the research findings, being enmeshed within the outcomes of this study.

## 5. Conclusions

Social media has moved from being a novel innovation to an essential component in how information is created and consumed, and perspectives are crafted and shaped. This analysis shows that Instagram is populated by contrasting views on endodontic treatment and demonstrates how this variation might be confusing for consumers searching for information. The use of a multimodal methodology provides insights into the different professional and non-professional styles and strategies of communication. The semiotic mechanisms employed by influencers that help to drive engagement in their content could be employed more broadly by those wishing to counter misinformation relating to any aspect of oral health and dental care. Space should be made within dental curricula to assist students in critically assessing contemporary health communication strategies employed by influencers to help arm them in better counseling patients who may be exposed to such semiotically influential misinformation narratives.

The nature of professionally produced misinformation, where accepted knowledge is sensationalised and blended with untruths, is particularly concerning regarding its potential to mislead the public. Much of the professionally derived misinformation was also disparaging to the ‘mainstream’ profession, casting aspersions in relation to commercial conflict of interest and lack of patient care focus. Meaningfully addressing the behaviour of dental professionals who engage in the promotion and promulgation of misleading narratives needs a collaborative approach. Professional dental associations need to collaborate with regulators to take a compassionate approach to those who are often stigmatised and ostracised from professional groups. In taking this approach, constructive discourse can be better developed, whilst proactively protecting patients from professionals who spread misinformation.

## Figures and Tables

**Table 1:** Themes and sub-themes.

Overarching Themes	Sub-Themes (Second Round)	Sub-Themes (First Round)
**Presentation of Root Canal Treatment**	A flawed treatment	*I.* *Root canal treatment as dangerous*
		*II.* *Ineffectiveness of treatment*
	2. Causation vs. correlation	*III.* *Link between endodontics and ill-health*
		*IV.* *Cancer*
	3. Sensational information and language	*V.* *Soft language of support*
		*VI.* *Parasites and pathogens*
**Presentation of Expertise**	4. Evidence-based arguments	*VII.* *Legitimacy of expertise*
		*VIII.* *Misuse of evidence*
	5. Commercial conspiracy	*IX.* *Commercial conspiracy against health*
		*X.* *Commercial interest in disparaging root canal treatment*
	6. Personal risk	*XI.* *Professional dissent and repercussion*
	7. Expertise and engagement	*XII.* *Semiotics of expertise*

## Data Availability

The raw data supporting the conclusions of this article will be made available by the author on request.

## Appendix A

### Appendix A.1. Adapted from Kress and Van Leeuwen [22]

The Three Metafunctions of Social Semiotic Analysis

(1) Representational Metafunction—who is depicted in the post? How are the individuals in the posts represented? In this metafunction, consideration is given to how a particular actor within the analysis is presented; for example, what about those individuals identified as experts (either dental or otherwise) invites the viewer to make the assumption of their role as a subject matter authority?

(2) Interactive Metafunction—how do posts encourage the viewer to interact with the actors within the text? Within this metafunction, consideration of contact, distance and points of view are important in understanding how the consumer of the video is intended to relate and consider the different actors features in the post.

(3) Compositional Metafunction—how do the other two metafunctions come together to create specific events? What is the overall message and portrayal in the post? In considering this metafunction, factors such as salience (what is most eye-catching within the video or post?) and modality (how similar is the text to reality?) are considered.

### Appendix A.2. Semiotic Example (Post 2)—Drdome1 (👍 1529 💬 33)

Dr Dome shares a reaction video of himself watching Luke Belmar being interviewed about his belief in the conspiracy theory relating to root canal treatment being unhealthy and being a professional conspiracy. The video begins with Dr Dome as a cutout at the bottom of the video, watching the video together with the viewer at the same time. As Belmar speaks, Dr Dome watches along nodding and smiling. At the end of the featured video of Belmar, the post cuts to a shot of Dr Dome speaking about the content of the video and the health problems that root canal treatments pose. The act of Dr Dome watching the video at the same time as the viewer helps to develop intimacy between the viewer and Dr Dome, with his reaction helping to give visual cues to the viewer as to how he feels about what he is watching.

In the video, Dr Dome wears a full white scrub suit, including a white scrub cap. The second part of the video, where Dr Dome is speaking to the camera, is shot in a dental surgery environment; in the background, there is an impression material dispenser on the wall, and a panoramic radiograph sits on display on a computer monitor. These items work with Dr Dome’s clinical attire to reinforce his clinical legitimacy and authority.

Throughout his dialogue, the camera jumps into close-ups with Dr Dome and out again several times, enhancing the feeling of intimacy. Dr Dome’s dialogue is also punctuated by graphics featuring the anatomy of teeth, featuring the dentinal tubules, suggesting that these are how bacteria enter root canal-treated teeth.

### Appendix A.3. Semiotic Example (Post 84)—Drgerrycuratola (👍 2485 💬 238)

Within this video post, Dr Gerry Curatola presents the dangers of root canal treatment in a clean, non-clinical space, in front of a large computer monitor which covers most of the wall. On the monitor is a picture of an abscessed tooth, accompanied by a radiograph of a root canal-treated tooth, which is annotated with markings pointing out “Infected Root Canal”. Dr Curatola wears a smart grey clinical scrub suit with his name and dental practice logo embroidered on the top. He wears glasses with thick blue, circular frames. He wears a gold-coloured watch.

He speaks in an authoritative and clear way, with the camera panning in and out as he speaks; when he comes to make important points, the camera zooms closer to Dr Curatola. Dr Curatola gesticulates as he talks, his language directive “You gotta listen-up and hear this”. His advice is unequivocal and certain that root canal teeth will lead to infection due to not being sterile and due to the fact that these teeth are dead. As the video finishes, Dr Curatola states, “The tooth, even if it’s not visible on a 3D scan, can be producing chronic inflammation that could be robbing you of your health.” As he states this, he is pointing at the camera. The messaging is one that directs the viewer to take personal responsibility for this important health issue.
